# Trends of online news media reported suicides in Ghana (1997–2019)

**DOI:** 10.1186/s12889-020-8149-3

**Published:** 2020-01-09

**Authors:** Tanko Abdulai

**Affiliations:** Department of Community Health and Family Medicine, School of Medicine and Health Sciences, University for Development Studies, Tamale, Ghana

**Keywords:** Suicide, Ghana, Online news media

## Abstract

**Background:**

Suicides have become headline news in most developing countries and often feature on front pages with accompanying graphic pictures on prominent news portals. There has been an increased reportage of suicides by the Ghanaian news media. This study aims to shed light on the trend of online news media reporting of suicides, and the epidemiology of media reported suicides in Ghana.

**Methods:**

An online search was conducted for news media reports of suicides committed in Ghana. One hundred and forty-two (142) news media reported suicides spanning 1997 to 31st July 2019 were retrieved and included in the analyses.

**Results:**

The victims of suicides were predominantly male (85.92%), young (mean age = 34.81 ± 15.71 years; range 10-86 years). Suicide by hanging (67.94%), the use of firearms (18.32%), and self-poisoning (8.93%) were the common methods used by the victims. There has been increased online news media reportage of suicides in Ghana in the last 3 years; 2017 witnessed the highest reportage of 30 (21%) cases. Marital or relationships and family problems were commonly cited as the reasons for the commission of suicides; mental disorders, and financial problems were also cited as immediate triggers of the suicides by close relations of the victims.

**Conclusion:**

There is an increased media reportage of suicides in Ghana, although this cannot be linked to a corresponding increase in suicide incidence. Interventions to decrease the risk of suicide among vulnerable populations (young adults) such as the identification of suicidal behavior, making mental healthcare services more accessible by integrating into the general healthcare service, public education, establishing a suicide hotline will be critical programs to help reduce suicide incidence in Ghana.

## Background

Suicide is the intentional killing of oneself. Globally, an estimated 800,000 people die due to suicide every year; this translates to one person every 40 s. Many more attempt suicide and this often is not disclosed, as suicide is deemed criminal in some countries. Suicide is the second leading cause of death among under 30 year olds globally and occurs throughout the lifespan. Suicide accounted for 1.4% of all deaths worldwide, making it the 17th leading cause of death in 2015 [1].

Suicide commission is usually attributed to social and psychological problems. Solitary living, extreme helplessness, hopelessness, and worthlessness, or defeat and failure, which may result from depressive psychopathology, are often linked to suicidal ideations and then to suicide [2]. Traumatic events, such as the loss of a close relation causes emotional distress and can lead to an enduring inability to cope with the loss. Financial or legal difficulties have been reported to cause emotional stress and sometimes leads to suicide commission. Physical illnesses such as chronic diseases like cardiovascular disease and diabetes have been linked with depression [3, 4]. Social factors such as a well-developed social support network, strong reasons for living, and responsibility for young children have been reported to be associated with a decreased risk of suicide, and the lack thereof comes with an increased risk of suicide [5, 6].

Several methods have been reported in the literature as means of suicide commission used by victims in ending their lives; perhaps the commonest are hanging, firearms, and self-poisoning in most countries [7–10]. Jumping from a height and charcoal burning are also common methods used in South East Asia. The choice of method is reportedly dependent on availability and the intended message the victim may wish to send. Men, however, generally use more violent means to end their own lives. In developed countries, suicide by firearms is more common compared to developing countries [11].

Suicide is still criminal in Ghana [12], and those who have attempted it have been prosecuted in the past. Annually an estimated 1500 persons commit suicide in Ghana [13]. There is, however, a paucity of data describing the epidemiology of suicide mortality in Ghana.

## Methods

There has been a recent increase in the news media reportage of suicide deaths in Ghana, this study; therefore, aims to shed light on the trend of online news media reporting of suicides, and the epidemiology of media reported suicides in Ghana.

### Data sources and coding

An online search of news reports of suicides committed in Ghana was conducted through www.google.com and www.bing.com search engines; additional searches were carried out from the websites of prominent online news sites in Ghana (www.ghanaweb.com; www.myjoyonline.com; citifmonline.com; www.adomonline.com; www.modernghana.com; www.graphic.com.gh; www.starrfm.com). A detailed list of sites from which reports were retrieved is presented in Additional file 1: Table S1. The search was carried out from 1st–31st July 2019. A combination of the words “suicide” and “Ghana” was initially entered, and the years 1995 through to 2019 were added; a specific year at a time, as “suicide Ghana 1995”, “suicide Ghana 1996,” and so forth. The google search returned over two million hits, but only the first ten pages were reviewed for possible inclusion. The headlines were read and only media reported suicide stories committed in Ghana were opened and the entire story read to abstract the reported date of the suicide, age of the victim, gender, occupation, reason for the suicide commission (as reported by family or close relation in the story), mode or means of commission of the suicide and whether victim left a note or not, and the link to the news item was saved for further review**.**

The analyses of the news media content were done in cognizance of the framework proposed by Klaus Krippendorff [14]. News reports meeting the inclusion criteria were reviewed to determine the main themes and patterns that resonate with the study objectives. A pattern emerged in terms of the socio-demographic characteristics, means of suicide commission, and causes or triggers. All reports were re-read to identify possible initial open codes; closed coding was then applied to meet the quantitative aspiration of this study (Additional file 1: Table S2). Some codes were further reduced to produce more statistically manageable data.

Abstracted data from the websites were entered into Microsoft excel 2013, data was then sorted according to date and entries carefully reviewed manually for duplicate stories. The date of the report, the name of the town and the demographic characteristics of victims were essential in spotting duplicates. Only one report was kept of identified duplicates (*n* = 19) for the analyses. To validate the abstracted data, a research assistant visited all the links from which data was abstracted and independently verified each record. A presentation of the method is included as Additional file 1: Figure S1.

### Inclusion criteria

All Ghanaian news media reported suicide cases committed in Ghana and available on online news media portals, with no suspicion of homicide in the report were included in the analyses. ***Exclusions***: Reports of attempted suicide (*n* = 25) and suspected homicide (*n* = 8) were excluded.

### Data analyses

The abstracted data was imported into Stata version 13 (Stata Corp. Texas, USA) for all statistical analyses. Counts with percentages and means with standard deviations were generated. Chi-square tests were performed to compare group differences in the mode of suicide commission and stated reasons for suicide commission.

## Results

**Fig. 1:**
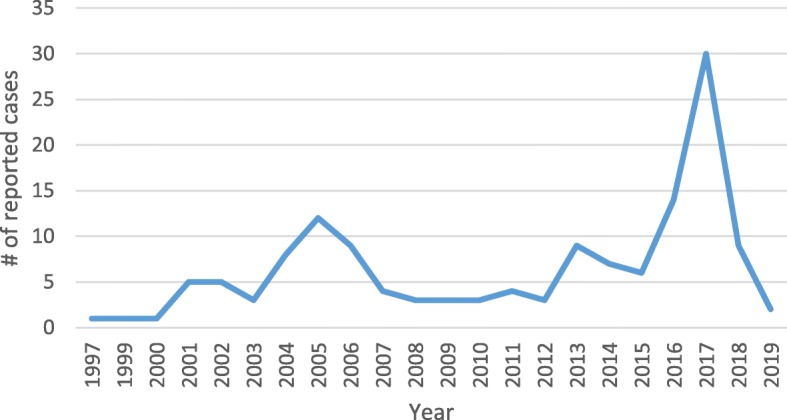
Suicide reporting trend in Ghana (1997–2019)

One hundred and forty-two (*n* = 142) online news media reported cases of suicides (1997 to 31st July 2019) in Ghana were retrieved and included in this analysis. Ghanaweb.com reported 60% of the suicides, followed by myjoyonline.com (8%). The year 2017 witnessed the highest reported suicide cases of thirty (Fig. 1).

The victims were predominantly male (85.92%), young (mean age 34.81 ± 15.71 years; range 10–86), female victims were comparatively younger (21.95 ± 9.32 vs. 37.43 ± 15.48, *p* < 0.001). The victims earned fairly good income (62.27%) deduced from their stated occupations. Eighteen (12.68%) of the suicide victims were students (55.56% were primary/Junior High School pupils). Greater Accra region, where all the online news media portals are located, reported the highest number of cases. The commonest mode of suicide commission was through hanging (67.94%), followed by firearms (18.32%) and through the drinking of poisonous substances (pesticides were commonly used) (8.93%). Marital or relationship problems (44.44%) were the most commonly reported reasons triggering the suicide commission, followed by reported mental problems or depression (18.06%) (Table 1). Financial problems (15.28%) were also commonly cited as reasons leading to the commission of suicides. Seven of the reported suicides were homicide-suicide, where the suicide victims (all men), had killed a spouse/family member/children before the commission of the suicide.

**Table 1:** Method of Suicide commission by baseline characteristics

		Method of suicide commission, *n* = 131(92.25)				
	Overall *n* = 142	Hanging 89(67.94)	Firearms 24(18.32)	Poison 11(8.840)	Other^a^ 7(5.34)	*P*-value
Gender						0.279
Male	122(85.92)	74(66.07)	23(20.54)	10(8.93)	5(4.46)	
Female	20(14.08)	15(78.95)	1(5.26)	1(5.26)	2(10.53)	
Reason						**0.005**
Marital/family/relationship problems	32(44.44)	15(51.72)	11(37.93)	3(10.34)	0(0.00)	
Mental/depression	13(18.06)	9(69.23)	0(0.00)	0(0.00)	4(30.77)	
Financial	11(15.28)	7(87.50)	1(12.50)	0(0.00)	0(0.00)	
Failed exam	5 (6.94)	4(80.00)	0(0.00)	1(20.00)	0(0.00)	
Other	11(15.28)	7(63.64)	3(27.27)	1(9.09)	0(0.00)	
Age mean(SD)	34.81 ± 15.71	33.90 ± 15.96	42.19 ± 15.45	29.14 ± 14.386	33.8 ± 16.26	0.2047
Income bracket						**0.050**
High	35(33.02)	22(64.71)	10(29.41)	2(5.88)	0(0.00)	
Average	31(29.25)	19(65.52)	7(24.14)	2(6.90)	1(3.45)	
Low	7(6.60)	5(71.43)	0(0.00)	0(0.00)	2(28.57)	
Student/Unemployed	33(31.13)	21(65.63)	3(9.38)	5(15.63)	3(9.38)	
Region						0.240
Ashanti	25(17.86)	15(62.50)	4(16.67)	4(16.67)	1(4.17)	
Brong Ahafo	5(3.57)	2(40.00)	1(20.00)	2(40.00)	0(0.00)	
Central	15(10.71)	10(76.92)	2(15.38)	0(0.00)	1(7.69)	
Eastern	23(16.43)	18(78.26)	3(13.04)	1(4.35)	1(4.35)	
Greater Accra	37(26.43)	19(59.38)	8(25.00)	1(3.13)	4(12.50)	
Northern	2(1.43)	1(50.00)	1(50.00)	0(0.00)	0(0.00)	
Upper East	6(4.29)	2(40.00)	2(40.00)	1(20.00)	0(0.00)	
Upper West	2(1.43)	1(50.00)	0(0.00)	1(50.00)	0(0.00)	
Western	14(10.00)	9(69.23)	3(23.08)	1(7.69)	0(0.00)	
Volta	11(7.86)	11(100.00)	0(0.00)	0(0.00)	0(0.00)	

^a^Suicide by jumping (*n* = 4), slitting/impalement(*n* = 2), and drowning (*n* = 1)

Although no significant difference was noted between the method of suicide commission and gender, men tended to use more violent means such firearms compared to women (23% vs. 5%). A significant difference in the choice of method of suicide commission and the stated reason for suicide was noted; generally, persons with stated mental disorders chose to jump from a high building and drowning compared to those with other stated reasons (*p* = 0.005). Age had no impact on the choice of method overall, but firearms were common among older subjects (*p* = 0.2047). A significant association between income status and method of suicide was observed; firearms death was more commonly used among high income earning subjects while hanging was more commonly used by those in the lower-income bracket (*p* = 0.050). No difference in the choice of method was noticed in the regions of Ghana; however hanging was universal in all the reported cases from the Volta region (Table 1). Age was noted to be significantly associated with the stated reason for suicide commission; older victims were more likely to have marital/relationship and financial problems attributed while younger victims had more mental health issues and failure attributed as the trigger for their decision to commit suicide (*p* = 0.010).

Nearly three-quarters of all firearm suicides (73.33%) were attributed to marital/relationship problems and all jumping and drowning were attributable to mental disorders (Table 2).

**Table 2:** Stated reason for Suicide commission by baseline characteristics

		Stated reason for suicide commission, *n* = 72(50.70%)					
		Marital/relationship problems 32(44.44)	Mental disorder 13(18.06)	Financial problem 11(15.28)	Failed exam 5 (6.94)	Other 11(15.28)	*P*-value
Gender							0.365
Male	122(85.92)	28(47.46)	9(15.25)	10(16.95)	6(5.08)	9(15.25)	
Female	20(14.08)	4(30.77)	4(30.77)	1(7.69)	2(15.38)	2(15.38)	
Suicide Method							**0.005**
Hanging	89(67.94)	15(35.71)	9(21.43)	7(16.67)	4(9.52)	7(16.67)	
firearms	24(18.32)	11(73.33)	0(0.00)	1(6.67)	0(0.00)	3(20.00)	
Poison	11(8.40)	3(60.00)	0(0.00)	0(0.00)	1(20.00)	1(20.00)	
Other	7(5.34)	0(0.00)	4(100.00)	0(0.00)	0(0.00)	0(0.00)	
Age mean(SD)	34.81 ± 15.71	**38.15 ± 16.82**	**30.92 ± 9.75**	**36.80 ± 10.61**	**19.60 ± 3.05**	**45.00 ± 15.42**	**0.010**
Income bracket							0.159
High	35(33.02)	7(36.84)	2(10.53)	3(15.79)	1(5.26)	6(31.58)	
Average	31(29.25)	8(53.33)	2(13.33)	3(20.00)	0(0.00)	2(13.33)	
Low	7(6.60)	1(33.33)	1(33.33)	0(0.00)	0(0.00)	1(33.33)	
Student/Unemployed	33(31.13)	6(37.50)	5(31.25)	1(6.25)	4(25.00)	0(0.00)	
Region							0.870
Ashanti	25(17.86)	2(15.38)	3(23.08)	3(23.08)	2(15.38)	3(23.08)	
Brong Ahafo	5(3.57)	3(100.00)	0(0.00)	0(0.00)	0(0.00)	0(0.00)	
Central	15(10.71)	3(57.14)	1(14.29)	0(0.00)	1(14.29)	1(14.29)	
Eastern	23(16.43)	5(41.67)	4(33.33)	2(16.67)	0(0.00)	1(8.33)	
Greater Accra	37(26.43)	8(44.44)	3(16.67)	3(16.67)	1(5.56)	3(16.67)	
Northern	2(1.43)	0(0.00)	0(0.00)	0(0.00)	0(0.00)	0(0.00)	
Upper East	6(4.29)	2(66.67)	1(33.33)	0(0.00)	0(0.00)	0(0.00)	
Upper West	2(1.43)	0(0.00)	0(0.00)	0(0.00)	0(0.00)	0(0.00)	
Western	14(10.00)	5(45.45)	0(0.00)	3(27.27)	1(9.09)	2(18.18)	
Volta	11(7.86)	2(50.00)	1(25.00)	0(0.00)	0(0.00)	1(25.00)	

## Discussion

There is an increased reportage of suicides in Ghana, although this cannot be linked to a corresponding increase in suicide incidence since official records of suicides in Ghana are not available in a centralized database. Causes of death in most cases in Ghana are often not reported, and a coroner’s report is not mandatory unless it is in suspected cases of homicide. These findings demonstrate that reported suicide cases have witnessed some increase over the last two decades, with the Greater Accra region where all the online news media portals are located recording the highest; Greater Accra may not necessarily have the highest incidence of suicide in Ghana but may be due to reporting bias. Most online news media do not have reporters across the country and will, therefore, report cases only within their geographic catchment area. Moreover, most online news media pick stories from print and radio reports from news outlets based in the capital. The proliferation of radio stations and increasing use of social media may partly contribute to the increase reportage of suicides in Ghana by online news media. There had been increased reportage on the general state of healthcare delivery and particularly the poor state of mental healthcare in Ghana during and after the 2016 general elections. Perhaps, the Ghanaian media were setting the agenda in their role to create awareness on the state of mental healthcare delivery in the country. The increased reportage of suicides around the same time the media were decrying the state of mental healthcare may fit into the agenda-setting and framing theories posited by communication scientists [15, 16]. Highlighting stories of suicides in the context of poor mental healthcare frame placed mental healthcare in the country at the forefront for policy consideration.

Evidence abounds that demonstrates that certain types of news coverage of suicide can increase the probable recurrence of the phenomenon (copycatting, contagion, or the Werther effect) in vulnerable groups particularly, among adolescents [17, 18]. This phenomenon may partly explain the increased reportage of suicides in the media particularly in the year 2017. The media reportage gave detailed explicit descriptions of suicides, the identity of the victim (such as name, place of work, school, etc.), and most reportages were sensational, some with accompanying photos of the victim. Victim identity protection is essential in an ethical media landscape.

There almost appear to be a four-year cyclical increase in the reporting of suicides in Ghana (2001, 2005, 2013, and 2017); these years coincidentally happen to be years after national elections in Ghana. The only exception was in 2009, the reasons behind this increase reportage after elections are still not clear, but may be attributable to disappointments following a defeat (no clear evidence exist to support this from the data).

**Fig. 2:**
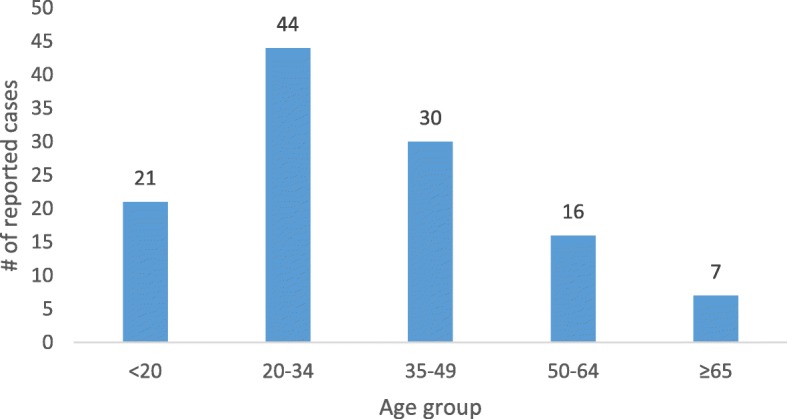
Number of reported suicides stratified by age

Forty-five percent of all reported cases were victims under the age of 35 years (Fig. 2). In previous studies by Reddy (2010), Turecki and Brent (2016), and Hawton and van Heeringen (2009), young adults were more likely to commit suicide compared to older adults [19–21]. Der et al. in a ten-year autopsy study of suicides in Ghana, made similar findings [22]. Young adults may have fewer reasons to live, and when they are troubled by circumstances that they cannot cope with, they are more inclined to committing suicides as a means of escaping from their troubles. Young adults are also more prone to suffer from depression [23] that may be triggers of suicide ideation and the subsequent committal of the act. In developing countries where mental healthcare is, least prioritized, troubled young adults are left to their fate with very little support to mitigate the effects of depression and other mental health challenges. The result of limited access to mental healthcare in developing countries further exacerbates the vulnerability of young adults to suicide ideation and to suicide committal.

Undoubtedly most suicide commissions are reportedly disproportionately higher in men compared to women, as noted in previous studies [9, 11]; the findings from this study present a similar gender distribution of suicide commission. Men are generally known to want to exhibit bravado in stressful situations. Even though women are generally reported to exhibit more depressive symptoms [23], they are better at coping because they are more inclined to share their problems with relations than men will. Men are also known to be affected severely when there is an economic downturn such as in the loss of employment, and this may put additional stress and lead to substance dependence. Substance dependence has been reported to be linked with suicides in population studies [19]. In the media reported suicides, the proportion of male victims reported to have committed suicide because of financial problems was twice that of female victims.

The method of suicide commission is usually linked to the method available to the victim and the message the victim intends to send. It is unsurprising that in the reported cases of suicides in Ghana, the commonest method used by victims was hanging with a nylon rope or bath sponge. Nylon rope and bath sponge are universally available in almost every home in Ghana. Suicide by hanging has been reported in many jurisdictions to be the commonest means by which victims end their lives [7, 9, 19], perhaps because this method is universally and readily available. Suicide by firearm is, however, more common in the USA compared to other countries due to the access of firearms in the USA [24]. Poisoning with agriculture chemicals has been reported to be more common among rural Chinese and other South-East Asian countries [10].

The predominant reason ascribed to the commission of suicides by close relations of victims was relationship related. Suspicion of infidelity and relationship termination (e.g., divorce or the threat of it, breakups, or estrangement), marital quarrels, and family quarrels. Adinkrah in a study on femicide-suicides in Ghana cited sexual jealousy, divorce, or the threat of it by female partners, and estrangements as the primary reported reasons to be the immediate triggers of femicide-suicides by close relations of male suicide victims [25]. Mental disorders were also cited by close relations of suicide victims as the immediate trigger of suicide; family members general stated that the victim was withdrawn, moody, or were behaving strangely. Reported mental disorders were more common in younger suicide victims compared to older victims. Most people who die by suicide have been known to have some form of psychiatric disorders, notably mood, substance-related, anxiety, psychotic, and personality disorders [3, 7].

The third reason ascribed for suicide by close relations of the victims was financial problems. The financial problems ranged from been indebted and unable to pay back, falling victims to scam or fraud (such as investment in Ponzi schemes that went bad), and losing nearly everything owned by the victims. The loss of financial wealth or the inability to meet one’s financial obligation can create enormous mental stress, and this has led some to suicide commission [2, 3]. More men and older adults were reported to have financial difficulties as triggers of suicide. Buron et al., in a study of reasons for attempted suicides from nine European countries, also cited financial difficulties among older adults (45-64 year olds), divorcees, and unemployed as one of the reasons attributable to the suicides of the victims [26].

The unavailability of comprehensive official sources of the cause of deaths in Ghana makes it challenging to estimate the incidence of suicide in Ghana. The findings here reflect only those reported by online news media portal, reporting bias will affect the extrapolation of this finding to the entire population. The media reports lacked autopsy reports to confirm the reported suicide cases. It is therefore likely that some suicide deaths were misclassified or went unrecognized as being a result of an accident or natural causes, and similarly, some natural causes or accidents could have been misclassified as suicides. A thorough screening of stories was, however, conducted to ensure the integrity of the reported suicide cases, where it was suspected to be a “foul play,” such reports were not included in the analysis. This study did not consider the ethical reporting of suicides, and whether reportage conformed to lay down guidelines, the study did not consider the effect of media reportage of suicides on subsequent occurrence (copycatting or the Werther effect) of suicides.

## Conclusions

There is an increased media reportage of suicides in Ghana. Most of the reported cases are in the national capital and the victims were predominantly men. Suicide by hanging is the commonest means, and immediate triggers attributed by close relations are relationships and family problems.

Intervention to decrease the risk of suicide among vulnerable populations such as the identification of suicidal behavior (withdrawal/isolation, aggression), mood changes (depression, anxiety, anger, irritability), making mental healthcare services more accessible (integration in the general healthcare service), public education, and establishing a suicide hotline will be critical programs to help reduce suicide incidence in Ghana. Programs on suicide prevention, such as screening for depression and counseling, should be developed and targeted to young adults in Ghana, especially in schools. Future studies should explore the ethical reporting of suicides and the effect of the reportage on subsequent incidence of suicides in the Ghanaian context.

## Supplementary information

**Additional file 1: Table S1**: Coding scheme for abstracted data. **Table S2**: Source of abstracted data. **Figure S1.** Flow chart of the Methodology (Adapted from *Krippendorff, 2004*).

## Data Availability

Primary Data sources are publicly available online from the archives of the news media in Ghana.
