# Supplementary material for: Trends of online news media reported suicides in Ghana (1997–2019)
Source: BMC Public Health. 2020 Jan 9;20:35. doi: 10.1186/s12889-020-8149-3 (PMC6953180; doi:10.1186/s12889-020-8149-3)
Supplement: Supplementary file 1 — Additional file 1: Table S1: Coding scheme for abstracted data. Table S2: Source of abstracted data. Figure S1. Flow chart of the Methodology (Adapted from Krippendorff, 2004). [file 12889_2020_8149_MOESM1_ESM.docx]

**Additional file**

Trends of online news media reported suicides in Ghana (1997-2019)

Author: Tanko Abdulai

Author information:

Department of Community Health and Family Medicine, School of Medicine and Health Sciences, University for Development Studies, Tamale – Ghana.

Correspondence:

email: [abdulai.tanko@uds.edu.gh](mailto:abdulai.tanko@uds.edu.gh) telephone: +233209105222

Table S1: Coding scheme for abstracted data

|  | **Code** | **Theme/ text** |
| --- | --- | --- |
| **Method of suicide** | Hanging | Hanged self |
|  | Firearm | Shot self to death |
|  | Self-Poisoning | Drunk poison/poisonous substance, acid, pesticide, weedicide, etc. |
|  | others | Jumped to death from a height, drown self, slit/slash wrist/throat |
| **Homicide suicide** | Homicide suicide | Killed partner, children, relatives and then killed self |
| **Demographic characteristics** | Age (numeric) | As reported |
|  | Gender (male/female) | As reported |
|  | Occupation | As reported |
|  | Income bracket | Deduced from the stated occupation: High-income earner= professionals such as lawyers, doctors, contractors, lecturers  Average income earner = other public and civil servants, small business owners, etc.  Low-income earner = unemployed, laborers |
| **Stated Reason for suicide** | Marital/relationship problems | Family dispute, Spousal/partner infidelity, abusive environment |
|  | Financial challenge | - debt, economic loss, failure to meet financial obligations such as school fees, provide for the family |
|  | Mental disorder/depression | withdrawn, kept to self, agitated, not behaving well |
|  | Others | Exam failure, team lost, failed ambition, terminal illness, |

Table S2: Source of abstracted data

|  | New platform | Number of stories retrieved | website | Online since | Type of content |
| --- | --- | --- | --- | --- | --- |
| 1 | Adom FM | 3 | http://adomonline.com |  | General news, radio |
| 2 | Citi FM | 10 | http://citifmonline.com |  | General news, radio |
| 3 | Ghana celebrities | 2 | https://www.ghanacelebrities.com |  | Entertainment news |
| 4 | Ghana Crusader newspaper | 1 | http://www.ghanacrusader.com |  | General news, print newspaper |
| 5 | Ghanaian times newspaper | 1 | http://www.ghanaiantimes.com.gh |  | General news, print newspaper |
| 6 | Ghana live | 1 | http://www.ghanalive.tv |  | Celebrity/entertainment news |
| 7 | Ghana reporters | 1 | http://www.reportghananews.com |  |  |
| 8 | Ghana web | 86 | https://www.ghanaweb.com | 1999 | General News, Classified ads and archives |
| 9 | Ghana news agency | 1 | http://www.ghananewsagency.org | 2012 | General News, news archive |
| 10 | Daily graphic newspaper | 2 | http://www.graphic.com.gh | 2010 | General news, print newspaper |
| 11 | Informafrica | 2 | http://www.informafrica.com |  |  |
| 12 | Justice Ghana | 2 | http://www.justiceghana.com | 2007 | Justice and Human rights issues |
| 13 | Modern Ghana | 6 | https://www.modernghana.com | 2005 | General news |
| 14 | Joy FM | 12 | http://www.myjoyonline.com | 1996 | General news, radio |
| 15 | News Ghana | 1 | https://www.newsghana.com.gh |  | Online business news |
| 16 | Northern Ghana | 1 | https://northernghana.net |  | Regional news |
| 17 | Peace FM | 3 | http://www.peacefmonline.com |  | General news, radio |
| 18 | Report Ghana news | 2 | http://www.reportghananews.com |  | Entertainment news |
| 19 | Starr FM | 2 | http://starrfmonline.com |  | General news, radio |
| 20 | The Herald newspaper Ghana | 1 | http://theheraldghana.com |  | General news, print newspaper |
| 21 | Ghana vibes | 2 | http://ghanavibes.com/ |  | Entertainment news |
|  |  |  |  |  |  |
| Total |  | 142 |  |  |  |

*All the media platforms have a national focus in the reportage of their stories unless otherwise stated under content

Reducing

Recording and coding

(142 Reports)

Narrations and Inferences

Analyzing

Exclusions:

1. 19 duplicates
2. 25 attempted suicides
3. 8 suspected homicide

Design and conception

Review of texts

Online search for suicides committed in Ghana

Abstracting and Unitizing data

Screening and Sorting

(194 relevant stories identified)

Figure S1: Flow chart of the Methodology (Adapted from *Krippendorff, 2004*)
